# Plasmonic Nanocomposites of ZnO-Ag Produced by Laser Ablation and Their Photocatalytic Destruction of Rhodamine, Tetracycline and Phenol

**DOI:** 10.3390/ma17020527

**Published:** 2024-01-22

**Authors:** Elena D. Fakhrutdinova, Anastasia V. Volokitina, Sergei A. Kulinich, Daria A. Goncharova, Tamara S. Kharlamova, Valery A. Svetlichnyi

**Affiliations:** 1 Laboratory of Advanced Materials and Technology, Tomsk State University, 634050 Tomsk, Russia; fakhrutdinovaed@gmail.com (E.D.F.);; 2 Research Institute of Science and Technology, Tokai University, Hiratsuka, Kanagawa 259-1292, Japan; 3 Laboratory of Catalytic Research, Tomsk State University, 634050 Tomsk, Russia; kharlamova83@gmail.com

**Keywords:** ZnO-Ag nanoparticles, plasmonic nanoparticles, pulsed laser ablation, photocatalysis, organic pollutants

## Abstract

Hydrosphere pollution by organic pollutants of different nature (persistent dyes, phenols, herbicides, antibiotics, etc.) is one of the urgent ecological problems facing humankind these days. The task of water purification from such pollutants can be effectively solved with the help of modern photocatalytic technologies. This article is devoted to the study of photocatalytic properties of composite catalysts based on ZnO modified with plasmonic Ag nanoparticles. All materials were obtained by laser synthesis in liquid and differed by their silver content and preparation conditions, such as additional laser irradiation and/or annealing of produced powders. The prepared ZnO-Ag powders were investigated by electron microscopy, X-ray diffraction and UV-Vis spectroscopy. Photocatalytic tests were carried out with well- known test molecules in water (persistent dye rhodamine B, phenol and common antibiotic tetracycline) using LED light sources with wavelengths of 375 and 410 nm. The introduction of small concentrations (up to 1%) of plasmonic Ag nanoparticles is shown to increase the efficiency of the ZnO photocatalyst by expanding its spectral range. Both the preparation conditions and material composition were optimized to obtain composite photocatalysts with the highest efficiency. Finally, the operation mechanisms of the material with different distribution of silver are discussed.

## 1. Introduction

Environmental problems associated with environmental pollution continue to remain not just a threat to the modern sustainable development of human civilization, but also to life on earth as a whole. The problem of polluting aquatic environments with various toxicants is still acute, and only a small part of the population has sufficient access to clean water resources [1]. One of the leaders in technogenic water pollution are complex organic compounds (OCs), textile and other dyes, antibiotics (especially those used in animal husbandry), pesticides and bacteriological pollutants [2,3,4,5,6,7]. One of the effective and environmentally friendly methods for purifying water from toxic organics is photocatalysis [8]. At this stage, the design, development and production of promising nanomaterials (photocatalysts) capable of decomposing various organic pollutants under the influence of light becomes a very important task.

Among the first materials that showed good efficiency in decomposing OC were wide-gap oxide semiconductors TiO_2_ and ZnO with *E*_g_~3.0–3.4 eV [9,10], which are still the reference materials for photocatalysis. In addition to their efficiency, they are easily accessible and have low toxicity. Thus, a large number of methods for their synthesis have been developed so far [11,12,13,14]. At the same time, ZnO has a number of advantages over TiO_2_ specifically in the decomposition of OC, since during the operation of the latter, instead of effective decomposition to simple products, photosorption and incomplete decomposition of persistent dyes often occur. For example, during photocatalysis of rhodamine B, the most effective process is not the complete destruction of the aromatic structure, but only N-diethelation [15,16]. The disadvantages inherent in wide-gap monophasic oxides associated with inactivity in the visible region of the spectrum and insufficient charge separation are leveled due to the creation of defects (doping and self-modoping) [17,18,19], or heterostructures [20]. Another effective approach to improve photocatalytic properties is the modification of the surface of semiconductor catalysts, such as ZnO, with noble metals having surface plasmon resonance (SPR) in the visible region of the spectrum (Ag, Au) [21]. The addition of a noble metal with a high electron yield work provides a negative shift of the Fermi level and the formation of the Schottky barrier, which significantly affects the separation of photogenerated charge carriers, whereas the excitation of the composite catalyst into the SPR band significantly increases the formation rate of photoinduced charge carriers [22,23,24]. In many cases, modification of ZnO NPs with metals such as Ag and Au was reported to significantly increase their photocatalytic activity in water purification from organic pollutants [25,26,27]. In the case of addition of Ag NPs, the increase in antibacterial activity of such nanocomposites was also reported [28]. It should be noted that when designing such composites with noble metals for practical application, it is important that the maximum photocatalytic and antibacterial effect is achieved at a relatively small level of additives.

Pulsed laser ablation (PLA) is rightfully considered one of the promising methods for creating modern nanomaterials for photocatalysis [29] and biomedical applications [30]. This method is cost-efficient, environmentally friendly, does not need complex precursors and is simple enough to produce complex structures, including nanocomposites [31,32]. The PLA method is excellent for producing ZnO, as well as increasing its photocatalytic properties, through modification with metals with surface plasmon resonance (SPR) to increase the spectral range of operation to the visible region [33]. Due to the suitable thermophysical characteristics of zinc, the power density of laser radiation and the productivity of ZnO preparation by PLA metal target in air are much higher than the productivity of obtaining TiO_2_ from Ti target [34,35].

There are various options for producing nanoparticles (NPs) in the ZnO-Ag system using PLA: (i) using metallic silver and (ii) using precursors (usually AgNO_3_). For instance, ablation of a silver target was carried out in a colloidal solution of commercially available ZnO [36], while others produced ZnO via PLA method too [37,38]. This approach was supplemented by irradiation of the resulting ZnO-Ag colloid with UV laser [39]. The second approach to the preparation of such metal oxide nanostructures involves the reduction of AgNO_3_ in the presence of ZnO NPs under the action of various radiation sources. Jung et al. carried out photoreduction of AgNO_3_ on the surface of ZnO NPs obtained by PLA under irradiation with light from a powerful Xe lamp [40]. In contrast, Whang and coworkers reduced AgNO_3_ by pulsed laser radiation of the second harmonic of a Nd:YAG laser on the surface of commercial ZnO NPs [41], while in [33] both the ZnO generation and its decoration via reduction were carried out by laser irradiation.

In this work, we used the metallic silver approach to prepare ZnO-Ag composite nanostructures. Both components of the composite particle were first obtained by laser ablation using nanosecond IR Nd:YAG lasers (1064 nm). The as-generated colloids were then mixed and thoroughly homogenized by means of ultrasound, after which several series of ZnO-Ag composites were prepared by further laser processing, post-preparation annealing or a combination thereof. In this work, for the first time, we used additional processing of colloidal mixtures based on Ag and ZnO NPs by focused laser radiation. This led to the formation of laser-induced plasma in the focal region of the lens, which provided conditions for more efficient interaction of the colloidal components and the formation of the ZnO-Ag interface with improved photocatalytic properties. The prepared composite catalysts were tested for the degradation of the persistent model dye Rhodamine B (Rh B), the widespread antibiotic tetracycline (TC) and the persistent organic pollutant phenol (Phen), all under low-power (0.3 W) irradiation of an LED source. The obtained results were analyzed from the viewpoint of morphology and composition, as well as surface properties of the newly reported catalysts.

## 2. Results and Discussion

### 2.1. Structure and Morphology of NPs

**Figure 1:**
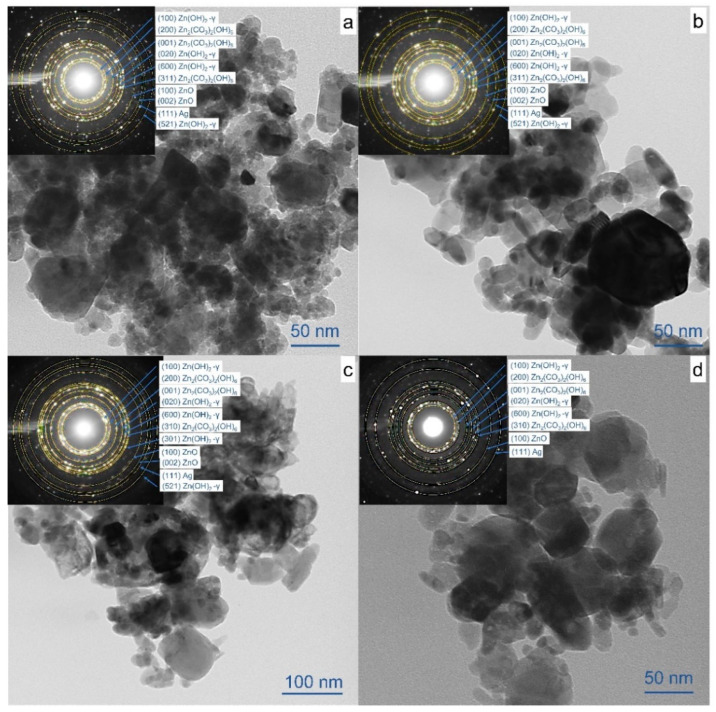
TEM images of samples (**a**) ZnO-1Ag, (**b**) ZnO-1Ag-400, (**c**) ZnO-1Ag-hν and (**d**) ZnO-1Ag–hν-400. Insets present corresponding SAED patterns.

The results of sample phase composition obtained by means of XRD are presented in Table 1 and Figure S1, while the morphology of prepared NPs is shown in Figure 1.

The initial ZnO sample (obtained by PLA of metallic Zn in water) is seen to have two phases: the dominant ZnO phase with the wurtzite structure (PDF Card # 04-008-8198) and an admixture of monoclinic phase of zinc hydroxycarbonate (PDF Card # 04-013-7572). Drying the as-produced colloidal solution in the air was found to lead to the formation of the so-called corrosion products of metallic Zn, i.e., zinc hydrocarbonate. This is known to occur through the interaction of zinc, oxygen and water, with the formation of zinc oxide and hydroxide, which react with dissolved carbonate species (CO_3_^2−^, HCO_3_^−^) with the subsequent formation of hydrogen carbonates [42]. The formation of Zn_2_(CO_3_)_2_(OH)_6_ during PLA in water was previously reported [43]. Additional laser treatment (ALT) is seen in Table 1 to lead to a decrease in the content of the hydroxycarbonate phase, as well as an increase in the crystallite size and a decrease in the specific surface area (see the adsorption-desorption isotherms of samples in Supplementary Materials Figure S2). Thermal treatment at 400 °C was found to lead to the decomposition of hydroxycarbonate (decomposition temperature of Zn_2_(CO_3_)_2_(OH)_6_ is about 260 °C, as seen in Supplementary Materials Figure S3) and the formation of 100% wurtzite phase, as well as a decrease in the surface area of NPs. This is similar to findings previously reported in [44]. According to XRF analysis, the Ag content in the samples was close to the claimed content and was the same for all four series of composite samples as they were prepared based on the same ZnO-*X*Ag series.

Addition of Ag was found to have almost no effect on the phase composition of the initially generated particles. At high Ag content ≤0.5 wt%, a reflex in the region of 37.5° 2***θ*** appears in XRD patterns which belongs to metallic Ag cubic syngony (PDF Card # 04-003-1472, Figure S1a). At the same time, silver was found to prevent particle enlargement during heat treatment, as the crystallite size of ZnO remains ~36–37 nm, while their specific surface area even increases slightly to 40 m^2^/g. ALT of mixed colloids obtained by PLA of Zn and Ag was also observed to prevent NP aggregation, while XRD patterns of samples with 0.5 and 1 wt% showed no peaks of metallic Ag. This implies a greater dispersion of Ag on the surface of ALT-processed ZnO (Figure S1) in comparison with their non-treated counterparts. Further heat treatment of this series of samples with low Ag addition did not lead to the appearance of metallic Ag peaks (Figure S1d).

The size and shape of the obtained NPs were examined by transmission electron microscopy (TEM). Figure 1a–d presents micrographs of composite particles with Ag content equal to 1 wt.%. The powders are seen to consist of agglomerated particles of irregular shape. In addition to the main fine fraction with the size of 5–30 nm, some larger particles as big as 100 nm are present (Figure 1a). After heat treatment at 400 °C, the smallest particles were found to grow bigger (Figure 1b) due to decomposition of hydroxycarbonates. ALT of the colloids is seen in Figure 1c, leading to some amorphization of the surface and formation of fused particles of irregular shape. Heat treatment of such samples at 400 °C did not result in a significant enlargement of particles, with their average size being mainly unchanged (Figure 1d).

Selected area electron diffraction (SAED) was used to clarify the structure of the obtained materials (Figure 1). For non-annealed samples, both SAED patterns and XRD analysis revealed phases of wurtzite ZnO (crystallographic plane (111)), zinc hydroxycarbonate Zn_2_(CO_3_)_2_(OH)_6_ (planes (200), (001) and (310)), and cubic metallic Ag (plane (111)). Also, the phase of unstable gamma zinc hydroxide—Zn(OH)_2_ (crystallographic planes (100), (020), (600), (521), PDF Card # 00-020-1437)—was found in non-annealed samples. SAED analysis of annealed samples (insets in Figure 1b,d) also show the presence of zinc hydroxycarbonate and zinc hydroxide, both phases not being detectable by XRD. This is probably owing to the formation of –CO_3_^2−^ and OH^−^ groups in the near-surface and surface layers during storage in air. The more blurred rings with a low content of even reflections in SAED pattern observed in Figure 1c (inset) also confirm some degree of amorphization of NPs caused by ALT, which is in good agreement with from TEM images.

**Figure 2:**
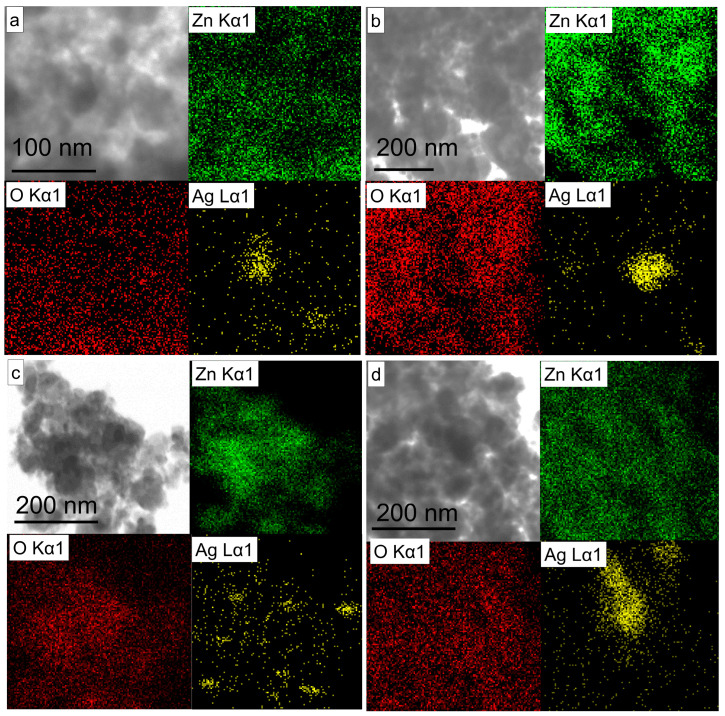
TEM image and EDS mapping of Zn, AgO and O in NPs: (**a**) Ag ZnO-1Ag, (**b**) ZnO-1Ag-400, (**c**) ZnO-1Ag-hν and (**d**) ZnO-1Ag–hν-400.

To analyze the distribution of silver in NPs, Figure 2a–d shows EDS mapping spectra of the elements Zn, O and Ag. For sample ZnO-1Ag, it is clear that silver is present throughout the sample surface, not only in the form of homogeneous small clusters, but also as relatively larger agglomerates (Figure 2a). After ALT processing, Ag is seen in Figure 2c to be more dispersed. Finally, heat treatment is seen in Figure 2b,d to lead to some enlargement of individual particles, while small clusters still remained present throughout the particle surface.

### 2.2. Electrokinetic Properties

**Figure 3:**
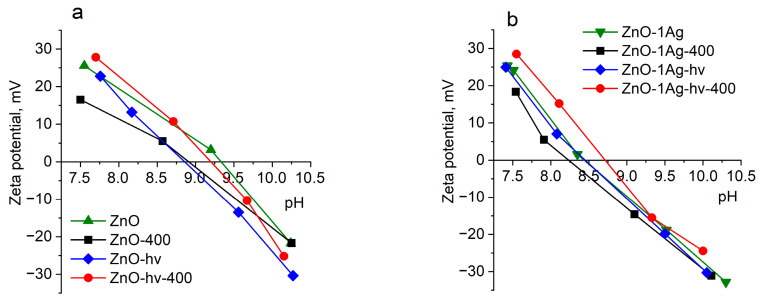
Zeta potential dependence on pH measured for sample (**a**) ZnO and composite sample (**b**) ZnO-Ag.

Figure 3 and Table 2 present the values of zeta-potential of particles dispersed in media with different pH. The zeta-potential of all analyzed powders is seen to be positive (from +16.5 to +28.5 mV), with the pH of their dispersions being around 7.5. Thus, based on the obtained results, it can be concluded that the surface state of the analyzed ZnO samples is similar and is largely determined by the Zn–OH/Zn–OH^2+^ and Zn-O-/Zn-OH equilibria on the particle surface, which is also in agreement with the SAED and SEM data. ALT of colloids does not appear to lead to significant changes in the electrokinetic properties of the particle surface (Figure 3a). Doping of NPs with silver was found to result in a small (about −0.5) shift of the isoelectric point (IEP) to the region of lower pH values. This indicates that the surface state of ZnO NPs does not change significantly upon its modification with silver (Figure 3b).

### 2.3. Optical Properties of NPs

**Figure 4:**
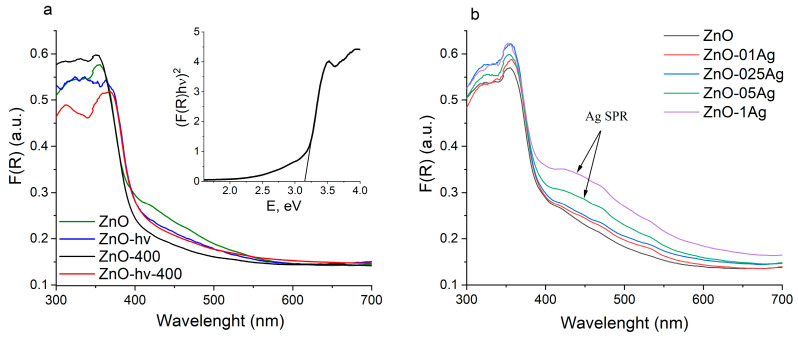
Diffuse reflectance spectra for NPs without Ag (**a**) and with Ag (**b**). Inset in panel (**a**) gives an example of *E*_g_ estimation.

UV-Vis spectra of powders investigated by diffuse reflectance spectroscopy (DRS) are presented in Figure 4, while the results of the band gap energy (*E*_g_) estimation are summarized in Table 1. Since the introduction of silver strongly affects the edge of the absorption band, *E*_g_ values were calculated by two methods: the classical Tauc method (Figure S4) and the derivation of absorption spectrum fitting (DASF) method (Figure S5) [45].

As seen in Figure 4a, the absorption band of the initial samples ZnO and ZnO-hν is in the region of 380 nm, corresponding to the band gap value of zinc oxide. The long-wavelength edge of the absorption band is diffused and extends into the visible region of the spectrum. This is owing to the presence of defects of different nature, most of which are related to oxygen vacancies and to interstitial zinc both in its ground and ionic states [46,47]. The absorption of zinc hydroxycarbonate is known to lie far in the UV range (*E*_g_ = 5.5 eV) and does not affect the absorption band edge of ZnO [48]. ALT of the colloid was found to have no significant effect on its absorption spectrum. Annealing at 400 °C of undoped samples leads to a slight long-wavelength shift, which can probably be explained by particle enlargement and the increase in their crystallite size (Table 1). There is no decrease in absorption in the visible region associated with the defective structure, as defectivity is preserved during annealing.

Figure 4b presents the absorption spectra of doped materials that were not subjected to additional laser treatment (spectra of the other samples are exhibited in Figure S4). When the amount of added Ag was 0.5 and 1 wt%, a characteristic shoulder in the region of 420–480 nm appears in the spectra, which is associated with the surface plasmon resonance (SPR) of silver. The SPR band is known to be sensitive to the size and shape of metallic NPs, as well as to the refractive index of the medium in which they are dispersed [49,50]. For small spherical NPs of metallic Ag, the peak of the SPR band is in the region of 390–420 nm, while for the ZnO-Ag composites, the Ag bands are strongly broadened and shifted toward higher wavelengths. This is believed to be due to the distribution of silver on the surface of zinc oxide and to the strong interfacial electronic interaction between Ag clusters and ZnO particles [51,52].

### 2.4. Photocatalytic Properties of NPs

#### 2.4.1. Photocatalytic Decomposition of Rh B

**Figure 5:**
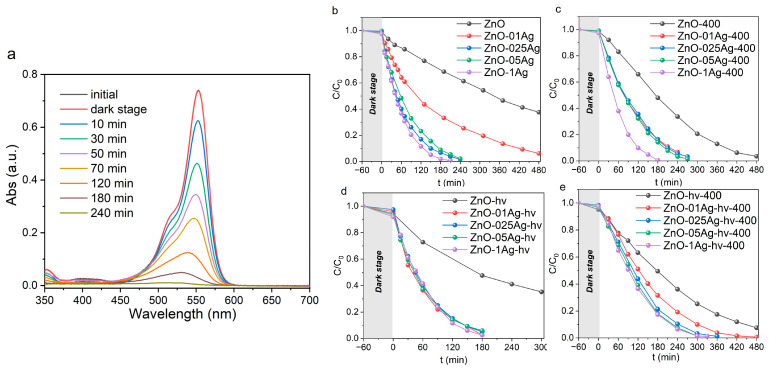
Absorption spectra of Rh B during decomposition in presence of sample (**a**) ZnO-025Ag. Decomposition kinetics curves of Rh B for series (**b**) ZnO-Ag, (**c**) ZnO-Ag-400, (**d**) ZnO-Ag-hy and (**e**) ZnO-Ag-hν. Irradiation: LED with λ = 375 nm.

The photocatalytic activity of samples was studied on the model Rh B dye irradiated by LEDs with wavelengths of 375 nm (soft UV-A) and 410 nm (visible region). Decomposition of Rh B was not observed under irradiation without photocatalysts (Figure S6a). Figure 5a shows how the absorption spectra of Rh B changed over time in presence of sample ZnO-025Ag irradiated with LED with λ = 375 nm. Under UV excitation, dye decomposition occurred with a slight shift of its main absorption peak (553 nm) to the short-wave region of the spectrum. This is related to the N-diethylation of Rh B and the formation of intermediate Rhodamine 110 [32,53]. Upon further irradiation, Rhodamine 110 is also effectively decomposed. The decrease in absorption in the entire visible range of the spectrum and discoloration of solutions indicates the destruction of aromatic rings of the Rh B structure. Kinetic decomposition curves for different series of samples are given in Figure 5b–e, and the rate constants are listed in Table S1. The initial powders of ZnO and ZnO-hν demonstrated relatively low photocatalytic activity (Figure 5b,d), which was due to the presence of zinc hydroxycarbonates on the surface of their particles. Annealing at 400 °C reduced the content of this phase, leading to an increase in the rate of Rh B decomposition. As a result, for both samples ZnO-400 and ZnO-hν-400, the long-wavelength band of Rh B completely disappeared after 8 h and the solution became discolored (Figure 5c,d).

The addition of Ag was found to lead to increased photocatalytic activity. For the initial series of samples without annealing and without additional laser treatment, the decomposition rate increases with increasing silver content in composite NPs (Figure 5b). At contents of 0.25–1 wt.%, complete Rh B decomposition was observed after 3–4 h. Additional annealing at 400 °C resulted in a decrease in the decomposition rate (Figure 5c). At Ag content of 0.1–0.5 wt%, complete decomposition was achieved in 4.5 h, while at an Ag content of 1 wt%, it was achieved in 3 h. For the series of samples with ALT, the decomposition rate is seen in Figure 5d to be almost independent of the Ag content, which is probably due to the increased dispersibility of Ag. All samples of this series were observed to degrade the dye within 3 h of irradiation. Annealing of such ALT-processed samples at 400 °C also reduced their photocatalytic efficiency (Figure 5e), which is probably due to the enlargement of Ag clusters dispersed on the surface of ZnO NPs.

**Figure 6:**
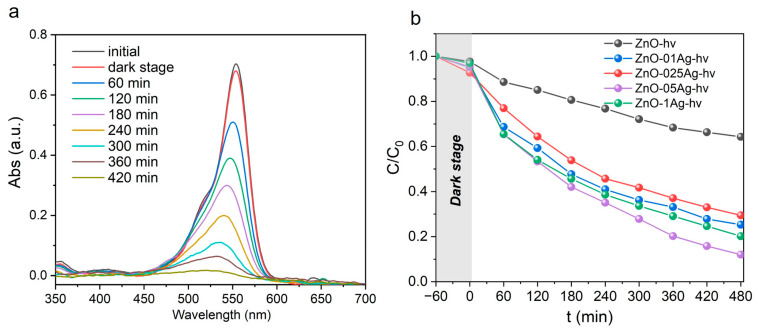
Absorption spectra of Rh B during decomposition in presence of sample (**a**) ZnO-1Ag-hν. Decomposition kinetic curves of Rh B for the (**b**) ZnO-Ag-hν series. Irradiation: LED with λ = 410 nm.

Figure 6a shows how the absorption spectra of Rh B (irradiated with LED with λ = 410 nm) changed over time in the presence of sample ZnO-1Ag-hν. The use of longer-wavelength blue radiation led to slower photodegradation of Rh B when compared with UV exposure and no complete decolorization of the dye was observed even after 8 h. Figure 6b shows kinetic dependences for a number of samples with different silver content obtained with ALT but without annealing. This series showed the best activity under visible light irradiation. The introduction of Ag significantly increased the photocatalytic activity of its NPs, similar to the case of UV light irradiation (Figure 5d).

**Figure 7:**
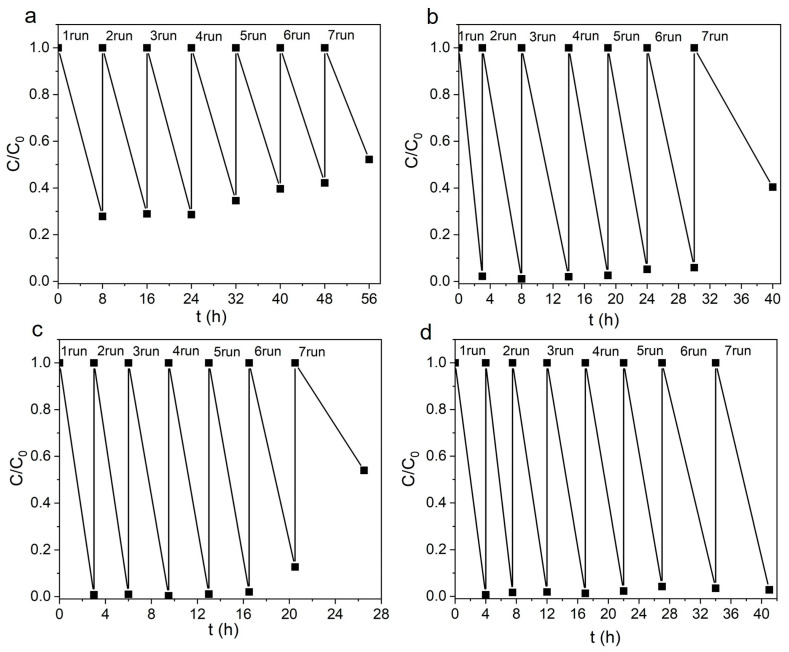
Cyclic stability curves of samples (**a**) ZnO, (**b**) ZnO-1Ag, (**c**) ZnO-1Ag-hν and (**d**) ZnO-1Ag-400 during decomposition of Rh B. Irradiation: LED with λ = 375 nm.

In addition to efficiency, photostability is also an important characteristic of the catalyst (Figure 7). Cyclic stability curves of NPs were measured under UV exposure at λ = 375 nm without removing the catalysts from the reactor. After each Rh B degradation cycle, 10–25 µL of concentrated dye solution was added to the reactor to restore the initial concentration (control was performed by the optical density of the solution). Figure 7a shows the cyclic stability curves for sample ZnO. Here, each cycle lasted 8 h, but no complete decomposition of the dye occurred (Figure 5b). The efficiency of the catalyst is maintained for three cycles, after which it begins to decrease. At the same time, the ZnO-Ag composite catalysts completely decolorized Rh B solutions. Sample ZnO-1Ag is seen in Figure 7b not to lose its efficiency for four cycles. The efficiency slightly decreased after cycles five and six (by 3–5%,) dropping by ~40% after cycle seven. Sample ZnO-1Ag-hν, which was ALT-processed, is seen in Figure 7c not to lose its efficiency during five cycles, after which it began to degrade rapidly. The best stability was shown by annealed samples. Figure 7d shows the performance for sample ZnO-1Ag-400, which demonstrated the best activity (Figure 5c). This photocatalyst is seen to work stably during all seven cycles and completely decolorizes Rh B. Its ALT-processed counterpart, sample ZnO-1Ag-hν-400, showed similar results.

**Figure 8:**
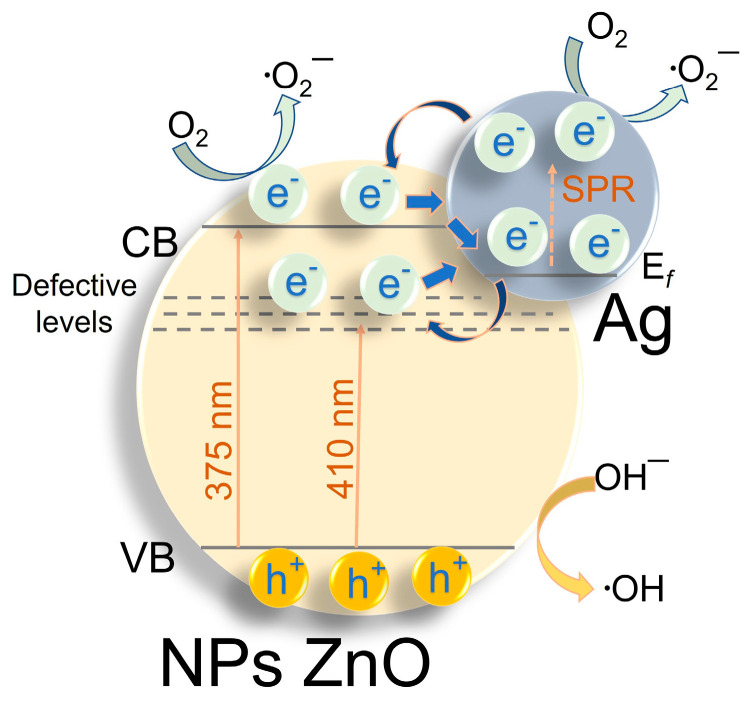
Schematic diagram of energy states and separation of electrons and holes in composite ZnO-Ag photocatalyst.

The influence of silver on the mechanism of ZnO-Ag photocatalyst operation can be presented in the form of the scheme of photogenerated charge transfers shown in Figure 8. Several processes of photogenerated electron transfer are possible at the interface between ZnO and metallic Ag. At UV irradiation λ = 375 nm, electrons are transferred from the conduction zone of ZnO to Ag clusters with a formation of the Schottky barrier, which is characteristic of most noble metals with high yield work [54,55]. Since PLA-prepared ZnO NPs have many defects of different natures (for example, oxygen vacancies and interstitial zinc atoms in their ground and ionic states [46]), such defects can act as either electron acceptors or hole traps. Therefore, the longer-wavelength irradiation (λ = 410 nm) can result in the transition of charge carriers from defective levels of ZnO to the levels of metallic Ag [56]. At excitation in the surface plasmon resonance (SPR) band, it is also possible to excite electrons of silver particles [57]. Such electrons can also participate in the generation of active particles involved in redox processes or migrate to the conduction zone of ZnO NPs. Electron transfer from the Fermi state (*E*_f_) of Ag NPs to defect levels of ZnO near the conduction zone is also possible. All the above-described processes with the involvement of silver NPs contribute to the enhanced photocatalytic efficiency of the composite nanostructures.

#### 2.4.2. Photocatalytic Decomposition of Tetracycline

**Figure 9:**
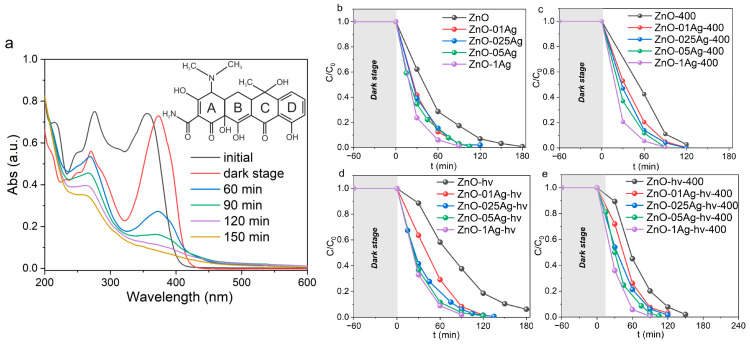
Absorption spectra of TC during decomposition in presence of sample (**a**) ZnO-05Ag. Decomposition kinetics curves of TC for series (**b**) ZnO-Ag, (**c**) ZnO-Ag-400, (**d**) ZnO-Ag-hν and (**e**) ZnO-Ag-hv. Irradiation: LED with λ = 375 nm.

The photocatalytic activity of ZnO-Ag NPs towards the decomposition of the antibiotic tetracycline (TC) was studied under LED irradiation with λ = 375 nm. TC is known to have two characteristic absorption peaks in the UV range located at 275 and 357 nm (Figure 9a). The shorter-wave peak at 275 nm is associated with the structure of aromatic ring A including enolic hydroxyl, amide and ketone groups, while its longer-wave peak at 357 nm belongs to the structure consisting of aromatic rings B, C and D [58]. Under irradiation without a catalyst, TC is stable and only ~6% of its molecules decompose within 8 h (Figure S4b).

Figure 9a shows the spectra of TC decomposition in the presence of sample ZnO-05Ag. Even at the dark stage of the experiment, when absorption-desorption equilibrium is established, a shift of the long-wavelength absorption band of TC from 357 nm to 375 nm is observed. This shift is due to the interaction of surface oxygen vacancies of ZnO NPs with numerous OH groups of TC molecules when they absorb on the catalyst surface. This interaction was previously reported to increase the degree of π-conjugation in the system, leading to a red shift of the absorption peak [59]. The photodegradation of TC in the presence of the catalyst results in a drop in optical density in both the 357 nm and 257 nm regions due to the decomposition of all aromatic rings of the molecule which are commonly designated as A, B, C and D (see Figure 9a). The obtained kinetic curves for all series of samples are presented in Figure 9b–e, and their corresponding rate constants are given in Table S2. In the presence of non-decorated ZnO NPs, complete degradation of TC was observed within 3 h. When composite ZnO-Ag NPs were used, the degradation rate increased along the silver content, so that in the presence of sample ZnO-1Ag, TC degraded after just 90 min (Figure 9b). Both ALT processing and annealing of ZnO-Ag powders at 400 °C were found to have practically no effect on their efficiency, as the average time of TC degradation was in the range 90–120 min.

#### 2.4.3. Photocatalytic Decomposition of Phenol

**Figure 10:**
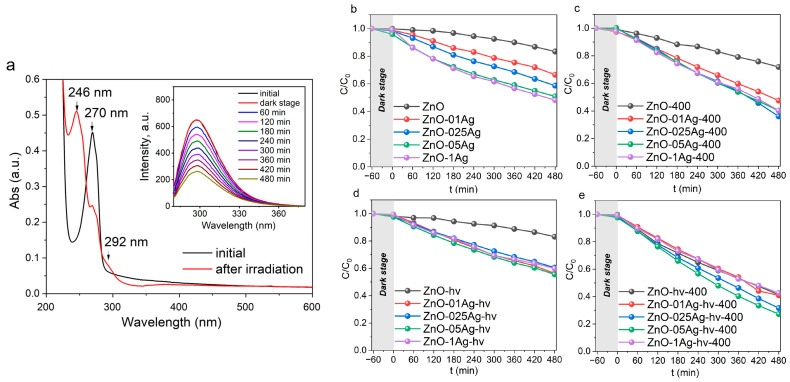
Absorption (and fluorescence, inset) spectra of Phen during decomposition in presence of sample (**a**) ZnO-1Ag-hν-400. Decomposition kinetics curves of Phen for series (**b**) ZnO-Ag, (**c**) ZnO-Ag-400, (**d**) ZnO-Ag-hν, and (**e**) ZnO-Ag-hν-400. Irradiation: LED with λ = 375 nm.

Phenol is a rather stable molecule that absorbs in the UV range of the spectrum shorter than 300 nm, which is why it is not decomposed when irradiated with LED radiation with λ = 375 nm without a catalyst (Figure 4c). Its photocatalytic decomposition in the presence of catalysts occurs with the formation of a number of intermediate products. Hence, after the photocatalytic reaction, its absorption spectra are seen in Figure 10a to show additional absorption bands at 292 and 246 nm that belong to the decomposition products hydroquinone and p-benzoquinone, respectively [32,60].

Since the absorption spectra of both the decomposition products and phenol itself overlap, this prevents the determination of phenol’s concentration. Therefore, the decomposition of Phen was determined from its photoluminescence spectra (inset, Figure 10a). After as long as 8 h of irradiation in the presence of catalysts, no complete decomposition of phenol was observed. The non-decorated sample ZnO and sample ZnO-hν showed the smallest photocatalytic efficiency, with decomposition efficiency in their presence being only 8–10% (Figure 10b,d). After annealing the catalysts, their efficiency was found to increase. As seen in Figure 10c,e, after 8 h of irradiation, 22 and 60% of Phen molecules were decomposed by samples ZnO-400 and ZnO-hν-400, respectively. The addition of silver to ZnO NPs increased the rate of Phen degradation for all series of catalysts. The highest photocatalytic activity was demonstrated by the ZnO-Ag-hν-400 series, which decomposed up to 70% of the ecotoxicant molecules after 8 h of irradiation (Figure 10e).

Table 3 compared the photocatalytic performance of our samples with that of ZnO-Ag nanocomposites previously reported by others and prepared by both laser-based and wet-chemistry-based methods.

The presented results indicate that the nanocomposites prepared in this study exhibited high photocatalytic activity when using relatively low-power radiation sources (LED, 50 mW) and a low loading of Ag (0.25–0.5 wt.%).

## 3. Research Methods and Material Preparation

### 3.1. Obtaining Materials Using PLA

Pulsed laser ablation of metal Zn (99.9% purity) Ag (99.99%) plates was carried out using the fundamental harmonic radiation of a Nd:YAG laser (LS2131M-20 model from LOTIS TII, Minsk, Belarus) with the following parameters: wavelength λ = 1064 nm, pulse duration 7 ns, frequency 20 Hz and pulse energy 150 mJ. At the beginning, two colloids were prepared separately by ablating the metal Zn target in 80 mL of distilled water for 30 min and Ag target in 80 mL of water for 1–5 min. The concentration of particles in the prepared dispersions was determined from the loss of target mass after ablation. The mass concentration of generated NPs (by metal mass) in colloids was ~300 mg/L (for Zn) and 10–30 mg/L (for Ag). Then the colloids were mixed in such proportions that the Ag content in the samples was 0.1, 0.25, 0.5 and 1 wt.% with respect to ZnO.

**Figure 11:**
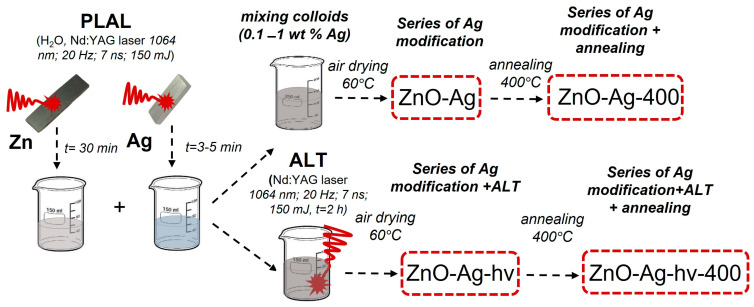
Schematic presentation of sample series and their preparation in this study.

A part of mixed colloids was sonicated for 15 min and then dried in the air at ~60 °C to a powder state. Below, this series of samples is denoted as ZnO-*X*Ag where *X* is the mass fraction of Ag. The sample without the addition of silver is designated as ZnO. Another part of mixed colloids was additionally irradiated with the same focused pulsed laser radiation as during their preparation. Such an additional laser treatment (ALT) of colloids was carried out for 1.5 h with constant stirring using a magnetic stirrer, after which the processed dispersions were also dried to a powder state. The use of a focused laser beam during ALT provided plasma locally generated inside the processed colloidal mixture [64], which stimulated the efficient formation of composite particles. This series of samples was denoted ZnO-XAg-hν where X is the mass fraction of introduced Ag. Similar to its non-irradiated counterpart, the sample without silver was denoted as ZnO-hν. In this way, two lines of samples, with and without ALT treatment, were obtained. Part of the material of the resulting powders was annealed in a muffle furnace at a temperature of 400 °C for 4 h. For heat-treated powders, the index 400 was added to the designation (for example, ZnO-05Ag-400). Schematically, the preparation of series of samples used in this study is presented in Figure 11. More details on material preparation and experimental setups, including the preparation of mixed colloids and ALT, can be found elsewhere [55,65].

### 3.2. Research Methods

The crystal structure of samples was studied using an XRD-7000 X-ray diffractometer (Shimadzu, Kyoto, Japan) with monochromatic CuKα radiation (1.54 Å) in the 2*θ* range of 20–90° and a scanning speed of 0.02 °/s using Bragg-Brentano geometry. Crystalline Si (*a* = 5.4309 Å, *λ* = 1.540562 Å) was used as an external standard to calibrate the diffractometer. The phase composition of collected patterns was analyzed using the PDF-4 database (Release 2022). To refine the parameters of the crystal lattice and determine the regions of coherent scattering (CSR), the full-profile analysis program POWDER CELL 2.4 was used.

The Ag content in the samples was estimated using an XRF-1800 sequential spectrometer (Shimadzu, Japan). To ensure accurate determination of low Ag loadings, preliminary calibration was performed.

The thermogravimetry analysis and differential scanning calorimetry (TG/DSC) were performed using an STA 409 PC Luxx analyzer (Netzsch, Selb, Germany) in a dry air atmosphere at a heating rate of 10 °C/min in the temperature range of 25–1000 °C.

Morphology and chemical composition of produced samples were also studied by transmission electron microscopy (TEM) using a JEOL JEM-2100 instrument (Tokyo Boeki Ltd., Tokyo, Japan) equipped with an energy-dispersive X-ray (EDX) analysis system at an accelerating voltage of 200 kV. Samples for TEM studies were prepared by depositing NPs dispersed in ethanol on copper grids coated with a carbon film.

Specific surface area and pore size distribution were determined by means of a TriStar II 3020 gas adsorption analyzer (Micromeritics, Norcross, GA, USA) using low-temperature nitrogen sorption. Before analysis, samples that were not subjected to heat treatment were degassed in a vacuum (10^−2^ Torr) at room temperature. Samples subjected to calcination were degassed in a vacuum (10^−2^ Torr) at 200 °C for 2 h using a laboratory degassing station or with a VacPrep Degasser (Micromeritics, USA) tool. The specific surface area was determined by the Brunauer-Emmett-Teller (BET) method.

Electrokinetic properties of dispersions were examined on an Omni S/N analyzer (Brookhaven, Upton, NY, USA). For this, powders of samples were dispersed in distilled water by means of sonicating for 6 min. The concentration of the prepared dispersions was 0.25 mg/mL. When studying the dependence of the zeta potential of dispersed particles on pH, the pH of the medium was adjusted by adding 0.1 and 0.001 M solutions of potassium hydroxide.

Optical properties of materials in the UV-Vis range were studied by diffuse reflection spectroscopy (DRS) on a Cary 100SCAN spectrophotometer with a DRA-CA-30I module (from Labsphere, North Sutton, NH, USA) in the wavelength range 230–800 nm. MgO powder was used as a reference for measurements. Then, the obtained DRSs were transformed using the Kubelka-Munk function, and hence the optical band gap of ZnO (as a direct-gap semiconductor) was estimated using the Tauck method from the curves plotted in the coordinates (F(R)hν)^2^ − *E*(eV). The calculated band gap obtained using the Tauck method was compared with the values obtained by the DASF method [36].

### 3.3. Photocatalytic Experiment

Photocatalytic activity of the prepared nanocomposites was assessed by the decomposition of the model dye Rhodamine B (with a concentration of 5 × 10^−6^ M), the broad-spectrum bacteriostatic antibiotic tetracycline (with a concentration of 5 × 10^−5^ M) and the organic pollutant phenol (with a concentration of 5 × 10^−5^ M). The concentration of tested catalysts was 0.5 g/L (15 mg of sample per 30 mL of aqueous medium, with no acid or alkali added). Before photocatalysis, a dark stage was carried out for 60 min to establish adsorption-desorption equilibrium. Then, the reactor was irradiated with LEDs with wavelengths of 375 nm (soft UV-A) and 410 nm (visible region). The wavelength of 410 nm corresponds to the surface plasmon resonance (SPR) of Ag NPs. The total radiation power incident on the reactor from the LEDs with 375 and 410 nm was 50 and 320 mW, respectively. The change in the concentration of Rh B and TC was monitored through their absorption spectra using an SF-56 spectrophotometer (OKB SPECTR LLC, Saint-Petersburg, Russia), while the changes in the concentration of Phen was determined from its fluorescence spectra using an RF-5031PC spectrofluorimeter (Shimadzu, Japan). To monitor the concentration of organics spectroscopically, at certain time intervals, aliquot samples were taken from the reactor, centrifuged (10 min, 12,000 rpm, 8 °C) and analyzed spectrally, after which the samples were returned to the reactor. Next, *C*/*C*_0_ curves were plotted versus irradiation time, where *C* and *C*_0_ are the current and initial concentrations of organic compound, respectively. Finally, the rate constants of the photocatalytic reaction were calculated from the corresponding kinetic curves, assuming the first-order reaction kinetics.

## 4. Conclusions

In this work, using laser techniques, four series of ZnO-Ag composite nanoparticles with silver content of 0.1–1 wt% were prepared. The composites were prepared from individual colloids first generated by pulsed laser ablation (PLA) of metallic Zn and Ag targets in water. According to X-ray diffraction, the as-prepared ZnO nanoparticles contained a zinc hydroxycarbonate phase Zn_2_(CO_3_)_2_(OH)_6_ as well as Zn(OH)_2_ hydroxide and the main wurtzite phase ZnO (>90 wt%). Additional laser treatment of the mixed colloids with focused laser irradiation allows for better dispersion of silver clusters on the surface of ZnO particles, while annealing at 400 °C destroys hydroxycarbonates in the samples. Nevertheless, some traces of Zn_2_(CO_3_)_2_(OH)_6_ and Zn(OH)_2_ are still seen in the SAED patterns of annealed samples. Adding silver was found not to affect the absorption band edge (and, consequently, the band gap energy) of the composite particles, and the broad SPR band of Ag clusters indicates a strong interfacial electronic interaction between Ag and ZnO.

At the same time, the addition of silver is shown to enhance both the photocatalytic properties of the composite particles and their stability as catalysts. This can be explained by the better charge separation in composite particles and by the changes in the kinetics of electrons due to the formation of the Schottky barrier and, possibly, the SPR effect in silver clusters. The obtained composite photocatalysts with low content of loaded Ag were demonstrated to effectively degrade the persistent model dye Rh B, as well as the well-known ecotoxicant phenol and the common antibiotic tetracycline (under low-power irradiation from an LED source with a wavelength of 375 nm).

## Figures and Tables

**Table 1:** Characteristics of samples.

Sample	Sample Composition		CSR *, nm	Ag Content, wt.% **	S_BET_ m^2^/g	Band Gap *	
	Phase	%				Tauc, eV	DASF, eV
Non-modified samples (as-prepared)							
ZnO	ZnO	90	37	–	36	3.29	3.31
	Zn_2_(CO_3_)_2_(OH)_6_	10					
ZnO-400	ZnO	100	43	–	21	3.25	3.27
Non-modified samples + ALT							
ZnO_hν	ZnO	95	62	–	23	3.11	3.28
	Zn_2_(CO_3_)_2_(OH)_6_	5					
ZnO-hν-400	ZnO	100	62	–	13	3.14	3.25
Ag-modified samples							
ZnO-01Ag	ZnO	90	36	0.13	40	3.30	3.31
	Zn_2_(CO_3_)_2_(OH)_6_	10					
ZnO-025Ag	ZnO	94	36	0.28	40	3.29	3.32
	Zn_2_(CO_3_)_2_(OH)_6_	6					
ZnO-05Ag	ZnO	93	37	0.49	40	3.29	3.32
	Zn_2_(CO_3_)_2_(OH)_6_	6					
	Ag	~1					
ZnO-1Ag	ZnO	93	37	0.98	40	3.30	3.32
	Zn_2_(CO_3_)_2_(OH)_6_	6					
	Ag	1					
Ag modification + annealing							
ZnO-01Ag-400	ZnO	100	40	0.13	18	3.25	3.26
ZnO-025Ag-400	ZnO	100	38	0.28	19	3.25	3.26
ZnO-05Ag-400	ZnO	99	35	0.49	21	3.24	3.26
	Ag	~1					
ZnO-1Ag-400	ZnO	99	35	0.98	26	3.25	3.27
	Ag	1					
Ag modification + ALT							
ZnO-01Ag-hν	ZnO	95	40	0.13	39	3.10	3.29
	Zn_2_(CO_3_)_2_(OH)_6_	5					
ZnO-025Ag-hν	ZnO	95	38	0.28	38	3.10	3.29
	Zn_2_(CO_3_)_2_(OH)_6_	5					
ZnO-05Ag-hν	ZnO	93	39	0.49	35	3.10	3.31
	Zn_2_(CO_3_)_2_(OH)_6_	7					
ZnO-1Ag-hν	ZnO	93	40	0.98	36	3.06	3.31
	Zn_2_(CO_3_)_2_(OH)_6_	7					
Ag modification + ALT+ annealing							
ZnO-01Ag-hν-400	ZnO	100	41	0.13	27	3.12	3.25
ZnO-025Ag-hν-400	ZnO	100	42	0.28	29	3.13	3.27
ZnO-05Ag-hν-400	ZnO	100	40	0.49	26	3.11	3.27
ZnO-1Ag–hν-400	ZnO	100	38	0.98	26	3.10	3.28

*: for phase ZnO, **: according to XRF data.

**Table 2:** Electrokinetic properties of samples.

Sample	pH_o_	ζ_o_, mV	pH_IEP_
ZnO	7.6	25.5	9.3
ZnO-400	7.5	16.5	8.9
ZnO-hν	7.7	22.8	8.8
ZnO-hν-400	7.7	27.8	9.2
ZnO-1Ag	7.4	25.3	8.4
ZnO-1Ag-400	7.5	18.4	8.2
ZnO-1Ag-hν	7.4	25.0	8.4
ZnO-1Ag-hν-400	7.6	28.5	8.4

**Table 3:** Photocatalytic properties of ZnO-Ag nanocomposites.

Synthesis Conditions	Parameters of Photocatalytic Experiment		Reaction Rate Constant for Best Sample	Refs.
	Pollutant, Concentration/Catalyst Loading	Light Source, Power		
Laser approach synthesis				
PLA Zn plate in H_2_O + PLA Ag plate in ZnO colloid (Nd:YAG laser, 1064 nm, 160 µs, 100 mJ)	Rhodamine 6G, ~10^−5^ M/ 2 mL of colloidal NPs	UV lamps (Sankyo Denki, Japan), 8 W, peak at 352 nm	0.0167 min^−1^ ZnO-Ag3 (Ag 23.4%)	[37,38]
PLA Zn plate in H_2_O (Nd:YAG laser (1064 nm, 7 ns, 90 mJ), calcined at 500 °C + photoreduction of Ag from AgNO_3_	Lindane (C_6_H_6_Cl_6_) 5 × 10^−5^ M/0.5 g/L NPs	UV–vis xenon lamp, 200 W	0.0352 min^−1^ ZnO/Ag (Ag~3%)	[40]
PLA Zn plate in isopropanol (Nd:YAG laser (532 nm, 7 ns, 25 mJ) + laser photoreduction of Ag from AgNO_3_ + calcined at 500 °C	Methylene blue (MB) 5 µg/0.15 g NPs	UV-Vis high-pressure sodium lamp	0.00547 min^−1^ 2 wt%Ag/ZnO (at pH 11)	[41]
PLA Zn plate in H_2_O + PLA Ag plate in ZnO colloid (800 nm, 90 fs, 3.5 mW, 1 kHz)	MB 10 mg/L/ 0.33 g/L NPs	250 W metal halide lamp (GE ARC250/T/H/ 960E40)	0.0419 min^−1^ 6 wt% Ag/ZnO at pH 10)	[49]
PLA Ag-coated ZnO target in H_2_O (Nd:YAG laser (1064 nm, 5 ns, 300 mJ)	MB 2.7 × 10^−5^ M/ ~0.1 g/l NPs	UV-vis Hg lamp, VIS 2.11 klx, UVA 0.2 mW/cm^2^, UVB 0.02 mW/cm^2^, UVC 0.08 mW/cm^2^	0.0233 min^−1^ Zn_1000Ag (0.32%)	[61]
PLA Zn plate in H_2_O + PLA Zn plate in H_2_O + ALT of mixed solution (1064 nm, 7 ns, 150 mJ)	Rh B 5 × 10^−6^ M Phen 5 × 10^−5^ M TC 5 × 10^−5^ M/ 0.5 g/L NPs	LED 375 nm, 50 mW	Rh B, 0.0209 min^−1^ ZnO-1Ag-hν Phen, 0.0019 min^−1^ ZnO-1Ag-hν TC, 0.0589 min^−1^ ZnO-1Ag-hν	This work
Other ways of synthesis				
Ultrasonic microwave-assisted method for ZnO + thermal reduction of Ag from AgNO_3_	Rh B, methylene orange 1 × 10^−5^ M/ 0.5 g/L NPs	500 W Xe lamp with a 400 nm cut-off filter	0.0431 min^−1^ ZnO/Ag (Ag~10%)	[62]
Microwave-assisted one-pot method of Ag/ZnO synthesis with thermal reduction of Ag from AgNO_3_	Rh B 2.1 × 10^−5^ M/ 1.5 g/L NPs	Xe lamp 300 W and AM 1.5 filter were used as the simulated solar light.	0.1732 min^−1^ Ag:ZnO (8:92)	[27]
Hydrothermal method for ZnO and further loaded via precipitation with Ag (photo deposition from AgNO_3_) and CDots	TC 6.8 × 10^−5^ M/ 1 g/L NPs	UV-vis xenon lamp, 150 W (300–780 nm)	0.03389 min^−1^ Ag/ZnO 0.0489 min^−1^ CDots/Ag/ZnO	[63]

## Data Availability

The data presented in this study are available upon request from the corresponding authors.

## Supplementary Materials

The following supporting information can be downloaded at: https://www.mdpi.com/article/10.3390/ma17020527/s1, Figure S1: X-ray diffraction patterns of a series of samples: (**a**) ZnO-Ag; (**b**) ZnO-Ag-400; (**c**) ZnO-Ag-hv; (**d**) ZnO-Ag-hv-400; Figure S2: Nitrogen adsorption-desorption isotherms for BET of samples: (**a**) ZnO-Ag; (**b**) ZnO-Ag-400; (**c**) ZnO-Ag-hν; (**d**) ZnO-Ag-hν-400; Figure S3: TG-DSC (Thermogravimetry-differential scanning calorimetry) curve of ZnO; Figure S4: Diffuse reflectance spectra of a series of samples: (**a**) ZnO-Ag; (**b**) ZnO-Ag-400; (**c**) ZnO-Ag-hv; (**d**) ZnO-Ag-hν-400; and estimation of the band gap using the Tauc method insert; Figure S5: Example spectra for calculating *E*_g_ using the DASF method for sample (**a**) ZnO and (**b**) ZnO-hv; Figure S6: Absorption spectra of (**a**) Rh B, (**b**) TC and (**c**) Phen before and after 480 min LED λ = 375 nm irradiation; Table S1: Reaction rate constants for the decomposition of RhB under irradiation with LED λ = 375 nm; Table S2: Reaction rate constants for the decomposition of TC under irradiation with LED λ = 375 nm; Table S3: Reaction rate constants for the decomposition of Phen under irradiation with LED λ = 375 nm.

## Sample Availability

Samples of the ZnO-Ag photocatalysts are available from the authors.
