# Supplementary material for: Plasmonic Nanocomposites of ZnO-Ag Produced by Laser Ablation and Their Photocatalytic Destruction of Rhodamine, Tetracycline and Phenol
Source: Materials (Basel). 2024 Jan 22;17(2):527. doi: 10.3390/ma17020527 (PMC10818360; doi:10.3390/ma17020527)
Supplement: Supplementary file 1 [file materials-17-00527-s001.zip › materials-2795669-SI.pdf]

materials

Article

Plasmonic nanocomposites of ZnO-Ag produced by laser ablation and their photocatalytic destruction of rhodamine, tetracycline and phenol

Elena D. Fakhrutdinova [1,*], Anastasia V. Volokitina [1,2], Sergei A. Kulinich [2,*], Daria A. Goncharova [1], Tamara S. Kharlamova [3] and Valery A. Svetlichnyi [1]

[1] Laboratory of Advanced Materials and Technology, Tomsk State University, Tomsk 634050, Russia;
[2] Research Institute of Science & Technology, Tokai University, Hiratsuka, Kanagawa 259-1292, Japan
[3] Laboratory of Catalytic Research, Tomsk State University, Tomsk 634050, Russia

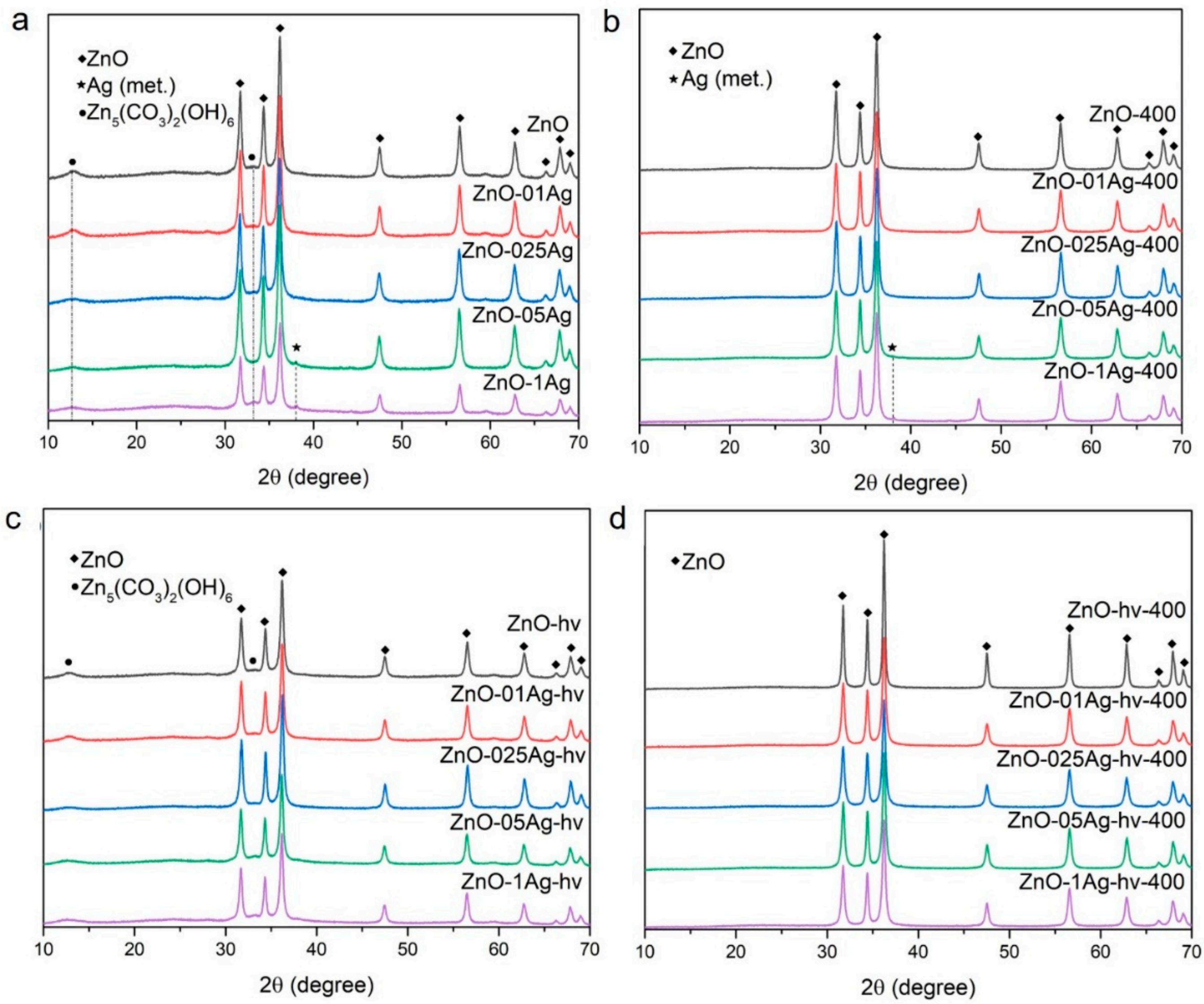

Figure S1. X-ray diffraction patterns of a series of samples: ZnO-Ag (**a**); ZnO-Ag-400 (**b**); ZnO-Ag-hv (**c**); ZnO-Ag-hv-400 (**d**).

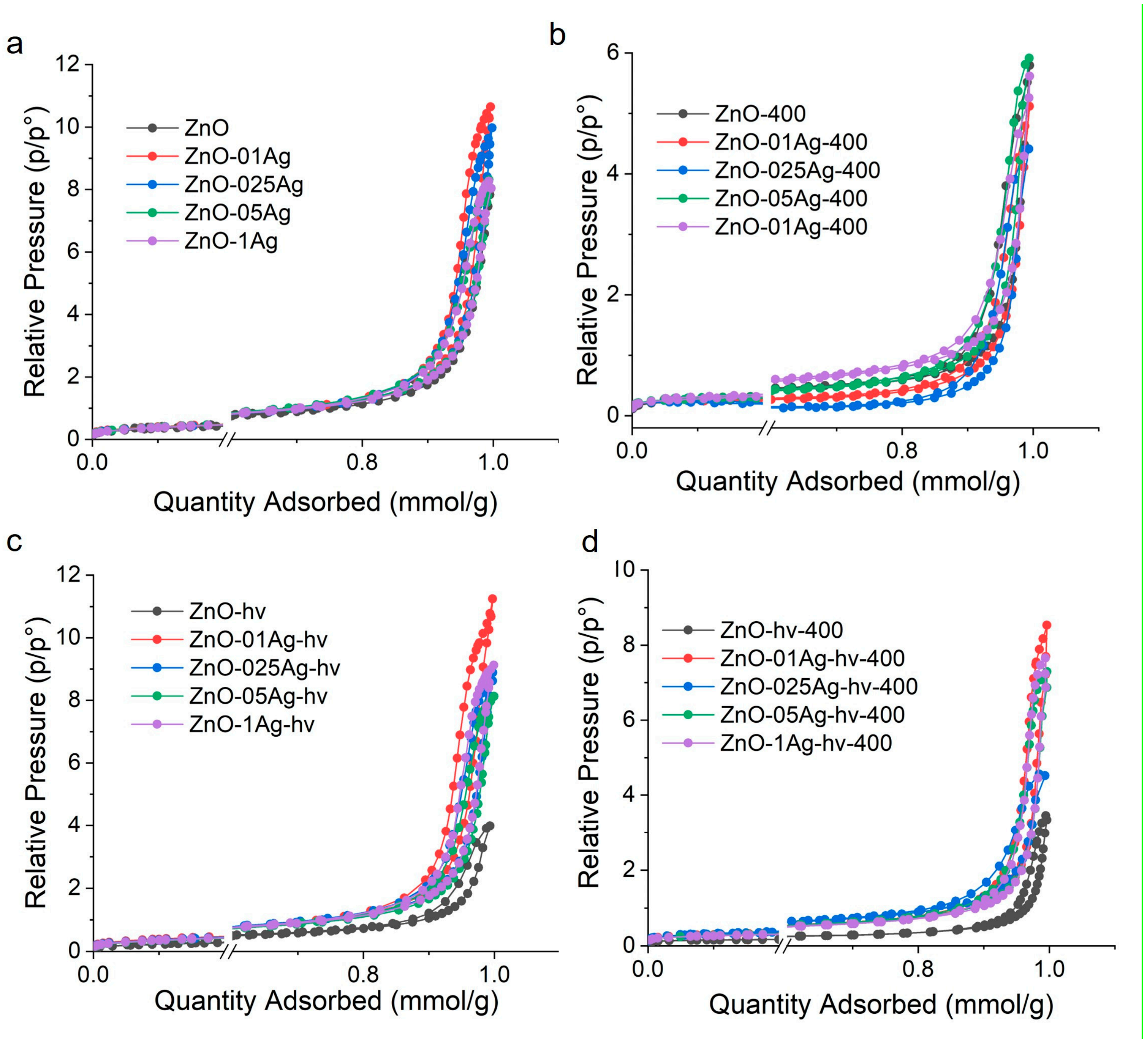

Figure S2. Nitrogen adsorption-desorption isotherms for BET of samples: ZnO-Ag (**a**); ZnO-Ag-400 (**b**); ZnO-Ag-hv (**c**); ZnO-Ag-hv-400 (**d**).

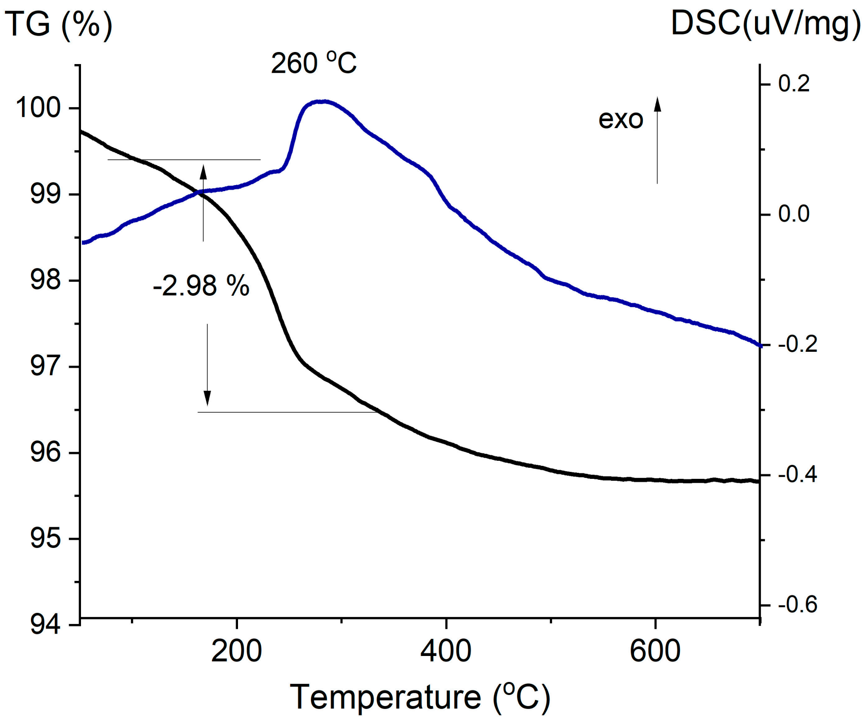

Figure S3. TG-DSC (thermogravimetry-differential scanning calorimetry) curve of sample ZnO

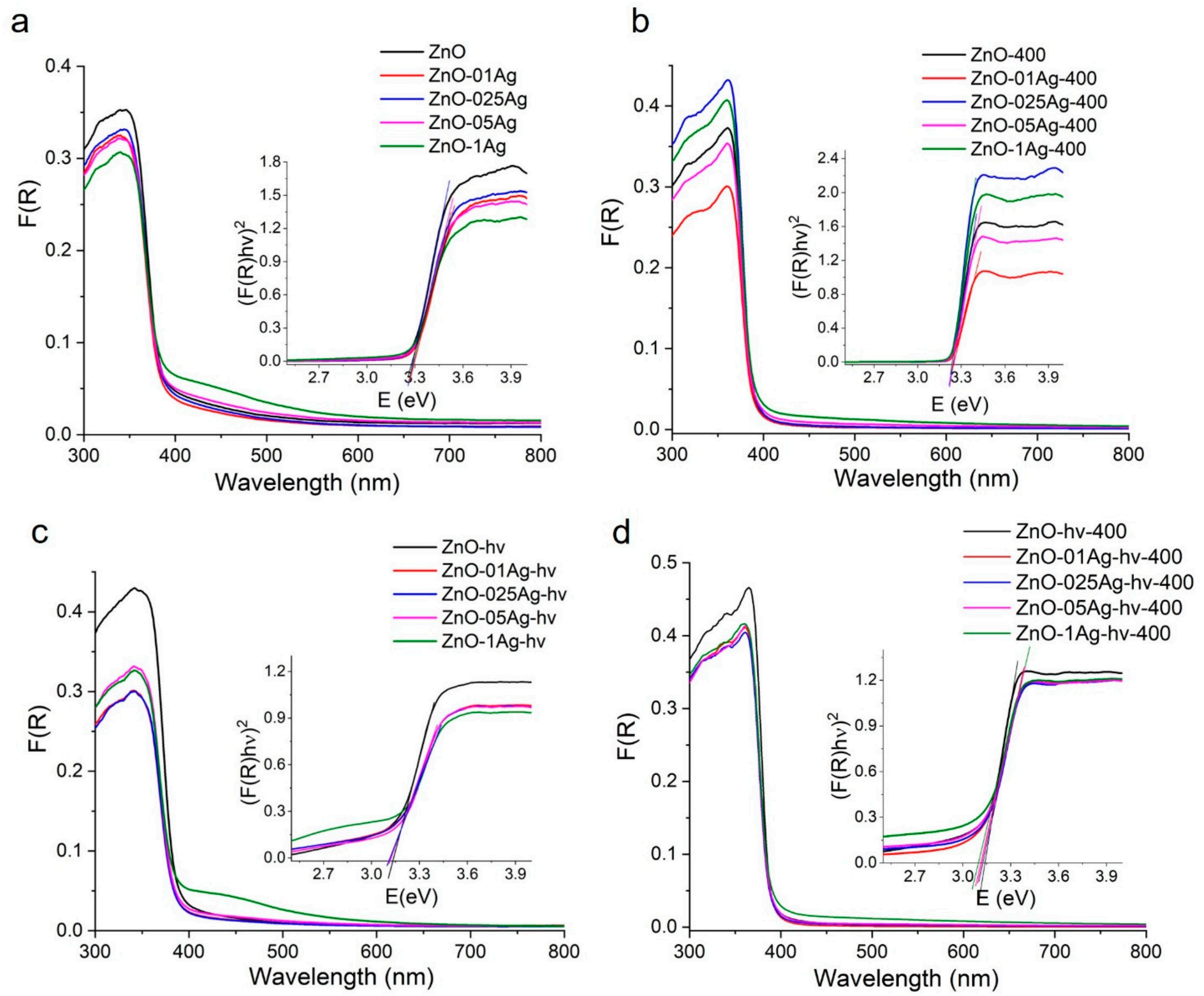

Figure S4. Diffuse reflectance spectra of a series of samples: ZnO-Ag (**a**); ZnO-Ag-400 (**b**); ZnO-Ag-hv (**c**); ZnO-Ag-hv-400 (**d**); and estimation of the band gap using the Tauc method (see inserts).

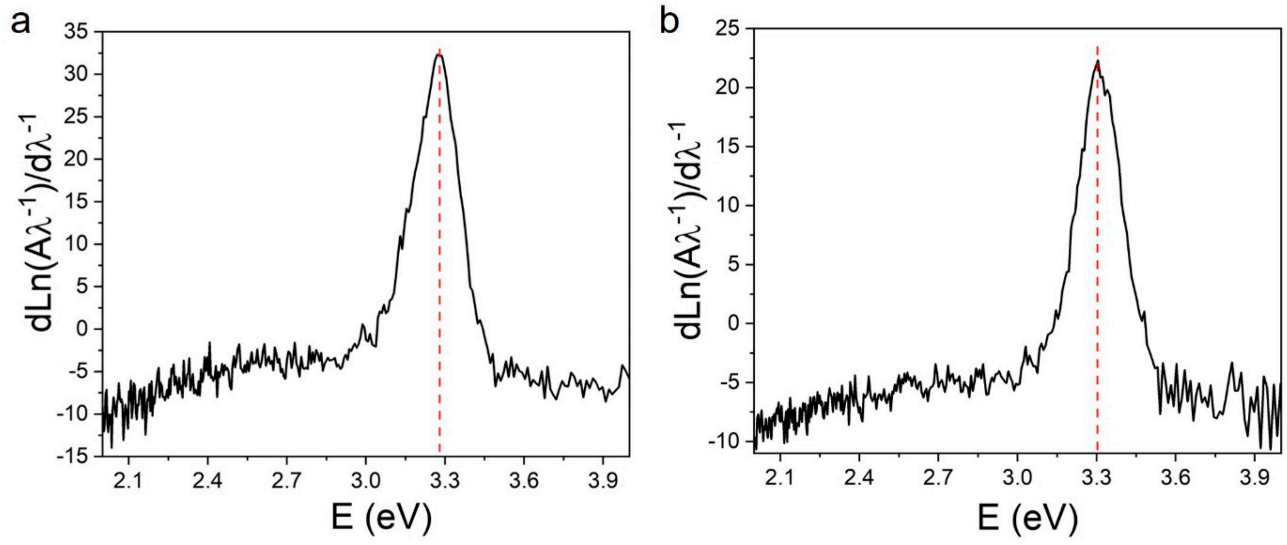

Figure S5. Example spectra for calculating E_g using the DASF method for samples ZnO (**a**) and ZnO-hv (**b**).

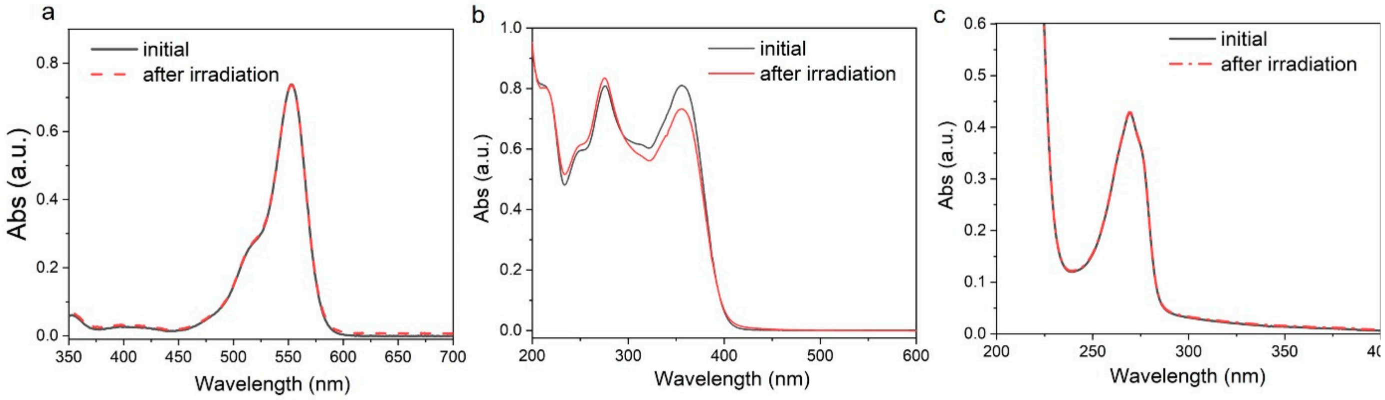

Figure S6. Absorption spectra of Rh B (**a**), TC (**b**) and Phen (**c**) before and after 480 min of irradiation with LED with λ = 375 nm.

Table S1. Reaction rate constants for the decomposition of RhB under irradiation with LED (λ = 375 nm).

Ag content, %	Reaction rate, min^{-1}			
	**initial**	**annealing**	**ALT**	**ALT+ annealing**
0	0.0017	0.0059	0.004	0.0051
0.1	0.0056	0.0115	0.0194	0.0080
0.25	0.0146	0.0122	0.0195	0.0108
0.5	0.0151	0.0123	0.0197	0.0108
1.0	0.0181	0.0209	0.0206	0.0121

Table S2. Reaction rate constants for the decomposition of TC under irradiation with LED (λ = 375 nm).

Ag content, %	Reaction rate, min^{-1}			
	**initial**	**annealing**	**ALT**	**ALT+ annealing**
0	0.0231	0.0294	0,0191	0.0312
0.1	0.0387	0.0333	0.0341	0.0356
0.25	0.0383	0.0358	0.0369	0.0392
0.5	0.0412	0.0362	0.0377	0.0449
1.0	0.0482	0.0589	0.0435	0.0558

Table S3. Reaction rate constants for the decomposition of Phen under irradiation with LED (λ = 375 nm).

Ag content, %	Reaction rate, min^{-1}			
	**initial**	**annealing**	**ALT**	**ALT+ annealing**
0	0.0004	0.0007	0.0004	0.0018
0.1	0.0008	0.0016	0.0011	0.0019
0.25	0.0011	0.0019	0.0012	0.0024
0.5	0.0013	0.0019	0.0012	0.0026
1.0	0.0013	0.0019	0.0011	0.0019
